# Paid homecare worker support for people living with motor neurone disease: A secondary analysis of people living with motor neurone disease and family member perspectives

**DOI:** 10.1177/26323524261452739

**Published:** 2026-07-04

**Authors:** Eleanor Wilson, Nicola Turner, Geraldine Macdonald, Christina Faull

**Affiliations:** 1 School of Health Sciences, University of Nottingham, UK; 2 University of Bristol, UK; 3 Dorothy House Hospice Care, Winsley, UK

**Keywords:** homecare workers, motor neurone disease (MND/ALS), secondary analysis, qualitative, family perspectives, patient perspectives, trust, emotional labour

## Abstract

**Background:**

People living with motor neurone disease (MND) increasingly receive complex, life-sustaining interventions at home, including ventilation, tube feeding, and cough assist support. These demands place substantial strain on family carers and often require input from paid homecare workers. Despite their essential role, little is known about how homecare workers contribute to complex MND care, how they integrate within multidisciplinary teams, or how families experience their involvement.

**Objectives:**

To examine people living with MND and family members’ perspectives of homecare worker roles, responsibilities, relationships when complex interventions are required.

**Design:**

A qualitative secondary analysis of data from two prior studies exploring home ventilation and tracheostomy ventilation in MND.

**Methods:**

Seven relevant NVivo nodes and 33 sub-nodes from interviews with 68 participants were re-coded using a deductive framework. Fourteen new nodes and 11 sub-nodes were generated and organised into three themes: care commissioning and provision; relationships; and the home environment.

**Results:**

Participants described fragmented and inconsistent care commissioning, requiring families to advocate persistently for adequate support. Challenges included funding barriers, high staff turnover, and limited MND-specific knowledge, which undermined trust and compromised safe, effective care. Relationships with homecare workers ranged from highly valued, stable partnerships to strained interactions shaped by competence concerns, emotional labour, and mismatched expectations. The presence of homecare workers and medical equipment transformed the home into a quasi-clinical space, reducing privacy, disrupting routines, and requiring households to adapt around care provision. Yet strong relationships with homecare workers could enhance quality of life.

**Conclusion:**

Homecare workers play a critical role in delivering complex home-based MND care, yet quality is inconsistent. Improving training, stabilising staffing, supporting care coordination, and preparing families for the relational and environmental impact of homecare are essential for fostering sustainable, trusted care relationships, and improving outcomes for people living with MND and their families.

## Plain language summary

Paid homecare support for people with motor neurone disease: Insights from people with MND and their families

This study explores how people living with motor neurone disease (MND), and their family members, experience support from paid homecare workers when complex medical interventions, such as ventilation, feeding tubes, suction and hoists are needed at home. As care needs increase, families often need help from paid homecare workers, yet no research has asked families about what this support is like. To address this, we re-analysed interview data from two earlier studies about living with home ventilation in MND, and developed three main themes: Care commissioning and provision: Participants described the process of securing a care package as confusing and often exhausting, with multiple organisations involved. Families frequently had to push to get the support they needed. Problems included shortages of trained staff, high staff turnover, and delays caused by complex funding rules. Relationships with homecare workers: Good homecare workers made a huge positive difference. Relationships with homecare workers ranged from highly positive, built on trust, skill and becoming ‘part of the family’, to strained, particularly when workers lacked MND-specific training or confidence with equipment. Trust was crucial: when present it eased pressure on families but when absent it increased stress and vigilance. Impact on the home environment: Having carers in the house, changed home life, reducing privacy, disrupting household routines, and living spaces became filled with medical equipment and ever-present homecare workers. Families described feeling like hosts in their own homes and sometimes having to manage the emotional labour of being polite or accommodating even when exhausted. Overall, the study shows that homecare workers play an essential role in complex MND care, but quality is inconsistent. Better coordination and support for families to manage these close relationships within the home are vital to reduce pressure on families and improve quality of life.

## Background

Motor neurone disease (MND)/amyotrophic lateral sclerosis is a progressive neurodegenerative condition that causes muscles to weaken and can also lead to changes in cognitive abilities including decision-making and behaviours. Though the disease encompasses a spectrum of presentations, individuals tend to experience significant impacts on daily activities such as walking, using their hands, speaking, swallowing, and eventually breathing. While progression and prognosis can vary, MND is life-limiting, with most individuals dying within 2–5 years of symptom onset. Care often involves input from a diversity of professionals, a range of equipment and technology, and home-based support, placing significant demands on both health and social care systems and family carers.^1–3^ The complexity and intensity of needs mean that the quality, coordination, and responsiveness of care are critical to maintaining dignity, autonomy, and quality of life for people living with MND (plwMND) and their families.^2^

Advancements in health technologies have enabled more plwMND to remain at home while utilising life-sustaining interventions such as invasive and noninvasive ventilation, cough assist devices, suction equipment, and enteral (tube) feeding. However, this increasing complexity of care delivered in the home places significant demands on family carers and usually necessitates additional support from paid homecare workers (HCWs). According to National Institute for Health and Care Excellence (NICE) guidelines [NG42], individuals with MND should have access to personal and practical care provided by workers who are familiar with their specific needs.^4^ Despite this, there remains a limited understanding of the everyday responsibilities of HCWs, their integration within the MND multidisciplinary team, a consistent framework for competency and training, and how they collaborate with family carers in the home environment.

The existing homecare literature in MND predominantly focuses on the experiences and burdens of family caregivers.^2,5–8^ Studies on wider care provision for plwMND in the United Kingdom highlight inequalities in the availability of specialist care, the need for better coordination of care, and the need for more timely access to equipment, while emphasising the importance of a multidisciplinary approach.^9–11^ However, the specific contribution of HCWs within this network of home support remains underexplored. Herber and Johnston^12^ provide a comprehensive overview of HCW roles in community palliative care, highlighting their involvement in personal care, emotional and social support, domestic tasks, respite provision, and collaboration with professionals and families. This review was updated by Forward et al. in 2024, excluding healthcare workers, concluding that HCWs were not adequately trained for the important role they play in advanced illness and feel isolated and unappreciated.^13^ Similar issues have been noted in dementia care, where HCWs face undervaluation of their role, significant emotional labour, and unmet training needs.^14–16^ A recent study on person-centred care for people with dementia by Xu et al.^17^ highlighted the importance of communication between HCWs, integration within the wider care team and navigating relationships with families to reduce tensions and enhance the contributions of HCWs. Families of older adults with dementia in Australia report the need for more flexible care and increased stress when HCWs did not have experience or dementia-specific knowledge.^18^

The specific demands of MND, such as progressive muscular deterioration, loss of mobility and speech, and reliance on complex medical equipment, require HCWs to possess specialised skills, and warrant particular attention. HCWs can play a vital role in providing respite for family carers and mitigating family caregiver burnout, but breakdowns in care often lead to unplanned hospital or hospice admissions.^19^ Conversely, consistent, high-quality homecare may enable plwMND to maintain autonomy over their care preferences, including place of care and death.^20^ The national shortage of trained HCWs in the United Kingdom, compounded by funding cuts and delayed reforms of social care, further exacerbates the specific challenges posed by MND.^21,22^ Moreover, the availability and quality of homecare can significantly influence decisions around life-sustaining interventions, delay hospital or hospice discharge, and impact quality of life. For example, evidence from tracheostomy ventilation studies in MND shows that inadequate homecare provision significantly delayed discharge and limited access to the intervention.^23,24^ In MND, there has been some exploration of recipients’ knowledge of and access to homecare,^9,25^ yet there has been no focus on how families living with MND experience HCWs in their homes.^26^

A deeper understanding of the roles, responsibilities, and challenges faced by HCWs and the families they work with is essential for improving training, support, and integration into multidisciplinary care for people with MND. This paper is based on the secondary analysis of data collected in two previous studies conducted by E.W. including N.T. and C.F. examining the use of ventilation at the end of life and experiences of living with tracheostomy ventilation (see Table 1). Although HCWs were not the focus of these studies, insights from plwMND and their families underscore the critical role of HCWs in maintaining quality of life, including at the end of life, and merit further examination (Supplemental Material).

**Table 1.** Primary study details.

Study characteristics	Study 1	Study 2
Study period	October 2020 to June 2024	June 2022 to June 2024
Study management	Wilson	Turner and Wilson
Title	Exploring end-of-life decision-making with patients with Motor Neurone Disease (MND) using home ventilation: The perspectives of patients and their families. (VentMND)	Understanding living with tracheostomy ventilation for Motor Neurone Disease (MND) and the implications for quality of life. (TVLife)
Aim	To explore end-of-life decision-making about the use of home mechanical ventilation for MND	To understand living with tracheostomy ventilation for MND
Participants	• People with MND dependent on ventilation to support their breathing, • And their family members, and bereaved family members of people with MND who have died with their ventilation in place or chosen to have it withdrawn	• People with MND with tracheostomy ventilation, • Family members and bereaved family members of people with tracheostomy ventilation for MND, • Health and social care professionals
Data collection and analysis	Interviews (in-person, online, telephone, and email), inductive thematic analysis	Interviews (online, telephone, and email), inductive thematic analysis Photographs and ecograms*,^27^
Study outputs	^28–33^	^34–37^

MND: motor neurone disease.

* Not utilised for secondary analysis.

## Objectives

1. To systematically explore plwMND and FM perspectives of the roles, responsibilities, and care relationships of HCWs with experience of supporting plwMND with complex interventions
2. To utilise data from two primary studies to understand how HCW provision can be optimised to improve outcomes for families living with MND where complex care at home is required.

## Design

Secondary analysis of qualitative data involves re-analysing existing qualitative data sets to answer new research questions or explore the original research questions from a different perspective.^38^ Heaton^39^ identifies this as ‘supra analysis’. Both primary studies utilised a constructivist interpretivist methodological approach and with data collection via semi-structured interviews. Original interviews, while guided by interview schedules, were flexible and encouraged the stories the participants wanted to tell. During iterative data collection and analysis in both studies it became clear that HCWs were an important element of care experiences, which could have considerable impacts for care coordination and quality. These data helped formulate the present study and develop this secondary topic. The secondary analysis was designed to refocus on more direct questions about HCW roles and the experiences of plwMND and their families, thereby maximising the use of existing resources and potentially uncovering additional insights from the data. While still an underdeveloped method, secondary analysis has been used in this way in previous studies.^5,40,41^

## Methods

For the purposes of this study, the term HCW is used for paid professionals who provide personal care and support to people in their home. It includes the terms domiciliary worker, care worker, and healthcare assistant. In the United Kingdom, plwMND have their individual needs assessed and may be able to access a fully or partially funded social services package of social care. As their needs progress, they may be referred for an assessment by ‘Continuing Healthcare’ (CHC) which pays for a package of care to cover both health and social care needs. CHC is funded by the National Health Service and is available for those who require ongoing care due to complex and rapidly changing health needs that demand a high level of skill to manage effectively.^42^ While most services are arranged by the NHS based on individual need, plwMND can also apply to have a personal health budget, which can allow greater control and choice over the healthcare services they access.^43^ Participants talked about the care they received through social care, healthcare, and personal health budgets.

Although the primary studies focused specifically on the use of ventilation in MND (see Table 1), many participants used other interventions such as feeding tubes, cough assist, hoists, and communication aids. However, this was not routinely collated data and the function level was not assessed or gathered during the initial qualitative studies. Having undertaken both the data collection and analysis for the two primary studies, authors E.W. and N.T. have full access to the data (see Table 1 for details of both studies and where to find further information on their individual data collection methods). E.W. and N.T. are also familiar with the context of these data and the people who shared their experiences.^39,44,45^

### Ethical approval

Ethical approval for this study was provided by the University of Nottingham, Faculty of Medicine and Health Sciences Research Ethics Committee (FMHS 01-1024). Ethical approvals for the primary studies were granted by: Study 1 – London-Dulwich Research Ethics Committee (21/PR/0252); Study 2 – Leicester South Research Ethics Committee (22/EM/0256). Consent gathered for both studies was gathered via an online form; however, a small number of participants were sent paper consent forms and chose to provide written consent in this way. The consent obtained included permissions to reuse anonymised data to support future research.

### Analysis

**Figure 1.**
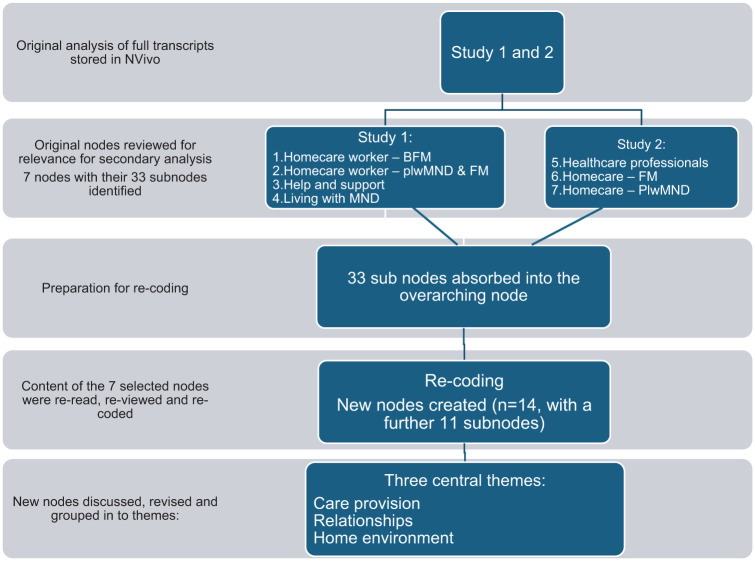
Process of secondary analysis.

As indicated above, it was clear from our original analysis that HCWs played a considerable role in the care of people with MND requiring more complex interventions. To explore this further we reviewed the nodes1 stored in the NVivo files for both studies^46,47^ with a deductive lens to explore the content and breadth in relation to the aims of the current study.^48^ This process is set out in Figure 1. We did not systematically re-code from all the full transcripts.^40^ Initially, E.W. and N.T. identified seven relevant nodes with 33 sub-nodes across the data sets of the two studies. These identified nodes had 594 separate codes assigned to them. These included nodes entitled ‘HCW’ and ‘homecare’ and their multiple sub-headings as well as broader nodes such as ‘help and support’, ‘healthcare professionals’, and ‘living with MND’. These nodes span data sets from 68 participants (in 64 interviews and 4 joint interviews) including bereaved family members (BFM; *n* = 30), plwMND (*n* = 19), and current family members (FM; *n* =1 9) across the two primary studies (74% of the total participants; see Table 2). This is not to say that the other participants did not have paid HCWs, just that they did not specifically talk about them during their interview.

**Table 2.** Number of participants included in secondary analysis per study.

Study	FM and BFM	PlwMND	Total
VentMND	5/10 (30/36 BFM)	11/16	46/62
TVLife	14/16	8/14	22/30
Total number of participants	49 (79%)	19 (63%)	68 (74%)

BFM: bereaved family members; FM: family members; plwMND: people living with MND; MND: motor neurone disease.

The seven relevant nodes were copied and pooled in a new NVivo file, while participants remained identifiable by their original study labels. All sub-nodes and their codes were absorbed (aggregated) into the overarching node to view the wider context and all content of the node. While data familiarisation and immersion had already taken place during initial analysis, coded sections were re-read with the lens of the new study and for re-familiarisation. Within the NVivo programme, all coded data could be expanded to view the section as situated in the full transcript as needed. This allowed us to then explore and reallocate these coded sections. A deductive, but evolving framework was used to re-code the original sections.^48^ This was based on some predefined codes derived from the new research questions, but with room to continue to draw out further codes from the data itself. This generated 14 new nodes along with 11 sub-nodes. Table 3 shows this new node frame. After comparison and discussion, similar nodes were grouped to create themes and larger nodes were reviewed to draw out greater nuance where appropriate. These were then reviewed, discussed, and refined by the research team (E.W., N.T., C.F., and G.M.) to ensure that they accurately represented the data.

**Table 3.** New nodes and sub-nodes generated and subsequent themes.

Node	Sub-node	Theme
Care systems and agencies	Care initiation	Care commissioning and provision
	Care management	
	Care systems and agencies	
	Type of care	
Coordination of need	Coordinator	
	Getting care in place	
Lack of HCWs		
Funding		
In-patient care		
Marker of deterioration		
Home		Home environment
Issues with HCWs		
Positive comments		Relationships
Relationships	With other HCPs	
Role		
Role of family member		
Vigilance		
Training	Knowledge of MND	
	Knowledge of equipment	
	Trust	Relationships
	Turnover	

HCW: homecare worker; MND: motor neurone disease; HCP: Healthcare Professional.

## Results

Three overarching and interrelated themes were revealed by this secondary analysis of data from plwMND and family members: care commissioning and provision; relationships with HCWs and their presence in the home environment.

### Care commissioning and provision

Participants described encountering significant challenges during the process of commissioning and procuring care packages. Commissioning arrangements varied geographically and according to the source and perceived availability of funding to meet complex care needs. A recurrent aspect of this was the necessity for families to act as advocates, frequently having to ‘chase, secure and fight’ for appropriate care packages. These packages, funded through disparate systems (e.g. NHS, local government social services), were sometimes deemed to be too expensive, making it difficult for local services to meet the clinical needs of the plwMND and their families:

The biggest stumbling block was getting funding from the social work department, but once we got that, my care company were very good; they reiterated we’ll take this on, we’ll train people. So it was waiting for funding, and that required letters to MPs, councillors and so on. That was a big battle . . .. It was just they felt it was too expensive a care package

**Because surely it must have been more expensive for you to be in [the intensive care unit] than . . .**

Exactly, that was the argument we were making, but of course the funding for [the intensive care unit] comes from a different source than social work. (Owen, plwMND, Study 2)

Care packages were often time-limited or subject to abrupt changes, with families reporting distress when trusted carers were replaced due to time-limited service contracts or budgetary constraints:

We had carers in then three times a day, and they were amazing, and they were council-run carers and they should have only been in for six weeks because they were a package that came in just for six weeks. And after the six weeks they said they’re going to put her on to a private company now and by this time she was being hoisted and the girls who were coming in, there were about ten of them, they were absolutely superb. They didn’t want to leave her, they said we want to stay – because we knew it wasn’t going to be long. So I fought tooth and nail to keep them and refused to have private carers and I knew they couldn’t just leave her. (Carol, BFM, Study 1)

The agency that had a contract with [Continuing Healthcare] . . . we’ve had three agencies providing packages . . . none of them have worked. . . . [plwMND] eventually said, ‘I need to do my own package, I’ll have my own personal [health] budget’. . . . That has worked in the main much better, simply because he can select his own staff, but it’s difficult to get the training they need for the tracheostomy. (Annie, FM, Study 2)

As Carol recounts, some HCWs demonstrated exceptional commitment to supporting families and the plwMND but were often powerless in the face of changes to funding and commissioning of care packages. Other families expressed some reluctance to ‘rock the boat’ for fear of jeopardising fragile care arrangements:

There’s often one carer on now [instead of two]. But the situation is that if [my parents] pushed it and said we can’t do it with one carer, that it might be a point at which they say well maybe this [care package] can’t exist anymore in the home situation, which would finish dad, and we’re all aware of that. So it’s do you rock the boat or do you accept what we’ve got? (Hannah, FM, Study 2)

Building a reliable team of HCWs was also dependent on their skills and abilities. Trust between families and HCW was significantly impaired when the latter lacked adequate knowledge of MND or had not received the training required to undertake the role safely and competently. In these circumstances, families found it difficult to maintain a good relationship with that HCW. As this BFM recalls, a demonstration of lack of basic knowledge from some care workers resulted in them continually negotiating with the care agency to request or refuse certain HCWs:

If I found somebody who I thought she’s nice, I can train her up, . . . [but] they didn’t know how to lift him, you know, they would do things like wash his bum and then use the same water to wash his face, and I was like ‘no you can’t’. Honestly as basic as that. I would ring the [agency] up. I think she took it personally. She used to say ‘I can’t please you’. And I said ‘well you could actually’. I would say so-and-so was really lovely today, can I keep her, can you put her in? No you can’t do that, we can’t do that – they’ve got to change them around. . . . they said I couldn’t refuse carers. I had to take what I was given. (Elizabeth, BFM, Study 1)

### Relationships

The presence of HCWs in the home introduced complex relational dynamics. The lack of private space and, in many cases, adequate space overall, had a significant impact on plwMND and their families. The physical and emotional intrusiveness of having care delivered in the home was often underestimated, particularly in cases involving complex care needs such as tracheostomy management, which generally requires two carers present 24 h a day:

I mean the main thing for me would be having carers there constantly because [plwMND] can’t have any privacy because they have to watch him constantly, because once [the ventilator] comes off he can’t breathe. And he needs suction and he needs cough assists and things like that. So it was just, I don’t know. It’s hard. . . ., I mean you make friends with them but they’re still strangers in your home. You can’t have a normal home life. . . . You lose a lot of, me and [plwMND] have lost, I think, a big part of our relationship . . . they’re just very intrusive. (Sue, FM, Study 2)

My carers are amazing people, some have been with me ten years, but it is difficult not having any real alone time. . . . It’s quite lonely always having people around you not really being able to behave like you want to. . . . It’s difficult to understand but when you have people with you all the time whose job is to make sure you are safe and happy it’s difficult to act sad or upset if you are feeling like that in front of carers. (John, plwMND, Study 2)

A small number of family members in Study 2 found having HCWs 24 h a day to manage the tracheostomy to be too much and chose to live elsewhere from the plwMND. PlwMND and family members reported challenges to their personal and marital relationships as well as their relationships with the multiple HCWs needed.

Positive relationships were often sustained through the ongoing negotiation of ways of coexisting within the home. When negotiations became strained, relationships could break down irreparably. Family members cited issues such as smoking, showing up unwell, spending excessive time on their phone, sleeping during night shifts, and lack of knowledge and skills as reasons for strained relationships with HCWs:

Mum was allergic to smoke, always had been, and they were arguing the point of well they’re allowed. They’re allowed to vape. And I said no they’re not allowed to vape in my property or outside my property. And it was all these bits where it became very fractious between them. By the end of it mum’s mental health started to deteriorate. (Dylan, BFM, Study 1)

Relationship strains were exacerbated when family members had little trust in the HCW’s abilities, and felt they had to remain close, and be on standby at all times. Some family members recognised that they and/or the plwMND struggled to accept HCWs in the home, wanting to maintain their role as sole carer:

Everybody says about the support for the carer and stuff, I didn’t get any if I’m being honest. But I think probably if I’m being fair that’s because I was probably quite unapproachable, didn’t want anybody else to do it. But [plwMND] was on suction because he was struggling to swallow. The only people he would let do the suction was me and [name] my daughter . . . I suppose it was that thing of he’s mine, don’t touch him! I want to look after him, I know what he likes. Because you just want to do everything for him. (Julia, BFM, Study 1)

Despite these challenges, strong relationships with individual carers were highly valued and contributed positively to quality of life:

If you have good personal care, if you have good carers, then quality of life can actually be pretty high; but if you have bad carers then it’s just horrendous. (Tess, FM, Study 2)

I was lucky to have had two private carers who worked for me about 6 months before my hospitalisation. They worked with me all through my time in the [intensive care unit]. They became experts in [tracheostomy ventilation] management. They have been able to train all the care company staff to our very high standards. Without ‘my team’, my care may have been compromised. A lot of care company staff have poor English competency which makes the care very challenging. (Max, plwMND, Study 2)

When there is trust in the HCW’s skills and abilities, the relief of burden on family members is tangible, allowing them respite from caring and enabling them to pursue quality of life:

People talk about having 24-hour carers in the house, not at all, they’ve become part of the family and they’re very good, so no . . . I’m able to leave him if we have good enough carers, I’m able to leave him and go out. (Pam, FM, Study 2)

Such relationships are complex and require considerable nurturing, communication, and mutual respect. As noted above, those HCWs considered to be excellent were the ones who went above and beyond. There were several reports of HCWs staying beyond the end of their shift to sit with plwMND at the end of life or to support family members. Becoming ‘friends’ with HCWs was perceived as a sign of a positive relationship:

And I think that, at the end when [plwMND] died there was just nobody. It just went from everybody to nobody. . . . And since, I think it was Christmas time, I sent a message to the carers sort of just to pop in because I had some little gifts. And . . . she was saying oh it’s strange having to ring the doorbell here. We’re so used to just walking round to the conservatory and walking in like it was home kind of thing. (Amy, BFM, Study 1)

Such relationships were vulnerable to disruption due to staff turnover and systemic constraints, underscoring the need for more stable and person-centred care models.

### Home environment

In addition to the constant presence of care staff, families highlighted the impact of medical equipment and storage requirements, as well as unfamiliar individuals accessing intimate areas of the home such as kitchens and bathrooms. These spaces, typically associated with privacy and personal significance, became shared and functioned as a workplace for HCWs, contributing to a sense of lost autonomy and emotional strain:

You couldn’t shift in here with stuff, everywhere, oh my god, I can’t tell you the stuff I threw away, how much went. . . . you had the carers in your house all that time and nothing’s your own and you’ve got no privacy . . .. (Madeline, BFM, Study 1)

If they don’t tidy up after themselves then I say I’m not here to tidy up. I’m happy for the night staff to cook in the kitchen, I’m happy for them to cook, but if they leave their pans and goodness knows what, I say I’m not your mother, you know. If you cook it’s absolutely fine but you have to tidy up after yourself. (Honor, FM, Study 2)

They’re all made welcome in my home to the point that I’ve just bought my sixth kettle in 12 years. I’ve had five microwaves and I keep saying if you can’t use it, if you don’t know how to use it, don’t use it, you ask someone, and if you use it you make sure it’s cleaned and dried out afterwards – and it doesn’t happen. All that sort of stuff is, that’s personal to me. (June, FM, Study 2)

The presence of HCWs during ‘out-of-hours’ periods heightened family members’ sense of intrusion. In the quote below, a family member describes the discomfort caused by this encroachment on their private space, noting that the HCWs’ presence left little opportunity to relax or behave as they would in their own home. This disruption to the normality of the home environment suggests that it is the household that must adapt to the HCWs’ presence, rather than the reverse:

We started off having care morning and evening, and then didn’t really like it because although it was helpful, it’s intrusive and they can’t come at the same time, for obvious reasons, and so you’re sort of dithering around and not knowing . . . So we’d got into this little routine of watching stuff a couple of hours in the evening, and with the carers coming at very variable times, that wasn’t very good. (Beth, BFM, Study 1)

The night carers coming in at 8 o’clock at night. . . . They’re coming in wanting a chat, how's your day been? And, you know, [plwMND]’s very polite, he will chat away to them but it’s exhausting. And things like, the feeling that you can’t go in and have a bath or a shower because these people are in your home. So you can’t go and just put your dressing gown on . . . you don’t want to be walking about your home like that. (Fiona, FM, Study 2)

In addition, the emotional and social burden expressed by many plwMND and family members when needing to maintain a level of politeness in front of strangers in their home was evident in many of their narratives. Their experiences highlight the tensions between necessary care and the disruption of personal space and routines.

## Discussion

This study highlights the complex and often fragmented nature of care commissioning and provision for plwMND and their families. Participants described significant obstacles to homecare commissioning and delivery, frequently shaped by funding limitations and disjointed organisational structures. A recurring element of this was the necessity for family members to assume the role of advocate, often needing to ‘chase, secure and fight’ for appropriate care packages. These packages, funded through disparate systems such as the NHS and local social care departments, were not always responsive to the clinical and emotional needs of plwMND and their families.^26^ Moreover, care arrangements were often time-limited or subject to abrupt changes, with families reporting distress when trusted carers were replaced due to contractual or budgetary constraints as perceived by plwMND and family members. Frequent turnover of HCWs emerged as a source of distress, potentially linked to systemic challenges in recruitment, training, and agency support. This churn was further compounded by decisions of plwMND and family members to dismiss or refuse HCWs they perceived as unsuitable.

As the participants illustrated, some HCWs demonstrated exceptional commitment and compassion, but many were limited by lack of appropriate skills or aptitude for complex care, leaving families feeling unsupported or hesitant to challenge existing arrangements for fear of destabilising fragile care arrangements. Limited knowledge of MND and a lack of training with unfamiliar, but essential, equipment further undermined trust in HCWs, particularly when compounded by high staff turnover.^18^

These findings highlight the fragmented and high-risk nature of care for extremely vulnerable plwMND. Aoun et al.^8^ describe how family carers often assume the role of care coordinators, navigating complex, and disjointed systems to secure appropriate support. This advocacy burden, echoed in our data, contributes to emotional exhaustion and undermines the sustainability of care arrangements. Findings from Study 1 showed that families managing home mechanical ventilation for plwMND experienced significant emotional and physical strain, often exacerbated by a lack of trust in the competence of professional caregivers and inconsistent support.^30^

Trust in HCWs emerged as a critical factor in both studies, influenced by HCW knowledge of MND and familiarity with essential equipment. Lisiecka et al.^49^ emphasise that trust is foundational to effective person-centred care in MND, enabling meaningful engagement and continuity of support. In the context of MND, the term trust refers to a specific form of confidence: trust that care workers have the competence to carry out essential tasks safely, and in some cases, to prevent or manage life-threatening situations.^26^ It is important to recognise this aspect of care, as it shapes both expectations and responsibilities within homecare arrangements.^50,51^ Clarifying who is responsible for conducting risk assessments within the care package is a key component of this form of trust, and appropriate training must be recognised as an essential measure for mitigating these risks and supporting safe, trustworthy practice. Trust sits at the heart of effective homecare relationships, shaping whether support feels enabling or burdensome for families. When HCWs demonstrate consistency, communicate openly, and show clear skills and competence, trust becomes possible, often transforming the experience of care.^49^ Where such trust exists, family members describe a tangible relief of burden, enabling them to step back from constant vigilance and reclaim aspects of their own quality of life. Meaningful trust meant they felt able to leave the house, confident their loved one was safe and well cared for. In this way, trusted HCWs do more than meet care needs: they provide the reassurance that allows families to breathe, rest, and live.

The presence of HCWs in the home introduced additional relational and spatial complexities. Pinto et al.^3^ and Trucco et al.^52^ report that family members experience ongoing emotional distress due to the progressive nature of MND and the transformation of the home into a clinical space. Across both studies families described the emotional and physical intrusiveness of home-based care, particularly in cases involving complex interventions such as tracheostomy management, which required round-the-clock support.^37^ The transformation of private spaces, such as kitchens and bathrooms, into shared, and often quasi-clinical environments, contributed to a sense of lost autonomy and emotional strain.^53^ The presence of HCWs in the household also introduced significant emotional and social burdens for plwMND and families. Participants described a loss of privacy and autonomy, with normal routines disrupted by the need to accommodate care schedules and the presence of outsiders. PlwMND and families frequently felt obliged to maintain politeness and engage socially with HCWs, even when exhausted or emotionally drained. This expectation to ‘perform’ within social norms adds an invisible layer of emotional labour, particularly for plwMND managing severe illness and for families juggling caregiving responsibilities.

Hochschild’s theory of emotional labour describes the management of feelings or ‘feeling rules’ to display socially or professionally appropriate emotions, often as a commodified part of paid work.^16,54^ Although family members are not commodifying their emotions in Hochschild’s classic sense, or in the way that HCWs themselves might be considered to be, they are exchanging emotional effort for the essential care required by the person living with MND.^16^ Previous work has focused on the emotional labour employed by family caregivers when caring for the ill person.^55,56^ Here we provide a novel lens on the element of emotional labour that is involved in accommodating the presence of outsiders, as this form of emotional management becomes an integral part of the extensive ‘work’ that families undertake when providing care at home.^57,58^ These dynamics created tension within the home environment, reinforcing the sense that households must adapt to care provision rather than care being integrated into family life.

Despite these challenges, strong relationships with individual carers were highly valued and positively influenced the quality of life for both plwMND and family members.^18^ As Bindley et al.^18^ note, the ‘quality of connection with care workers appeared to promote comfort and reassurance’ (p. 8). Family caregiver resilience and emotional wellbeing are often supported by positive interpersonal dynamics, even amidst the bureaucratic complexity, fragmented nature of services, funding inconsistencies, and understaffing that contribute to systemic shortcomings.^3^ However, these relationships were vulnerable to disruption due to staffing changes and systemic constraints, underscoring the need for more stable, coordinated, and person-centred care models.^2^

### Strengths and limitations

Qualitative secondary analysis provides an ethically sound and pragmatic approach to generating research insights on a sensitive subject from a small study population.^38^ Both plwMND and family carers were affected by fatigue and limited time for pursuing quality of life, therefore conducting a secondary analysis of existing data, rather than drawing again on the resources of a small study population, offered significant ethical and methodological advantages.^59^ We recognise that the primary studies were not designed to focus on experiences of having HCWs, their roles within the home or the wider multidisciplinary or palliative care teams.^60^ However, it is notable that such a substantial proportion of our samples raised issues around this topic in their narratives. As such we felt that it was appropriate to explore this substantial body of data with a new lens. This secondary analysis is part of an ongoing study which is also generating new data from HCWs and other key professional stakeholders. This will be reported elsewhere, however further details can be found on the study webpages: Complex Homecare in MND (CHiMND).

We also recognise that we used multiple forms of interview for data collection in both primary studies. These were designed to meet the communication needs of the participants.^61^ For example, data from plwMND gathered via email due to communication impairments was necessarily focused, with less opportunity for additional probes and queries; this may account for a lower contribution on this topic (63%) than that of family members (79%). Expanding the traditional qualitative interview approach may have influenced the type of data generated; however, we suggest there is no indication that this has compromised the credibility of the findings.

The focus of the original studies limits our ability to explore how, why, or when HCWs were introduced, and how they aligned with the wider multidisciplinary team. Importantly this paper does not capture the perspectives of HCWs or those involved in delivering care packages, representing a significant gap in understanding how to enhance the delivery of complex homecare.

## Conclusion

This study offers important new insights into the experiences of plwMND and their families who rely on HCWs, addressing a longstanding gap in the literature. By drawing on the perspectives of plwMND and their families, it reveals the way in which care is delivered within a fragmented, inconsistently commissioned, and frequently high-risk system. Families are often required to navigate complex organisational structures, advocate persistently for appropriate support, and manage the consequences of funding constraints, staffing instability, and variable levels of HCW knowledge. These pressures intensify the emotional and practical burden of caring for family members and HCWs, particularly in cases involving complex interventions for MND such as enteral feeding, home mechanical ventilation, or tracheostomy care. The study draws attention to the profound relational and spatial consequences of delivering complex care in the home. Families described the emotional and physical intrusiveness of having multiple HCWs in the home, loss of privacy, and the transformation of domestic space into a quasi-clinical environment. It is therefore essential that we understand these complex relationships and interactions so that we can better prepare families and HCWs, with the aim of fostering trusted and sustainable care relationships and improving the quality of care for all involved within the constraints of our current care systems.

## Supplemental Material

sj-docx-1-pcr-10.1177_26323524261452739 – Supplemental material for Paid homecare worker support for people living with motor neurone disease: A secondary analysis of people living with motor neurone disease and family member perspectives Supplemental material, sj-docx-1-pcr-10.1177_26323524261452739 for Paid homecare worker support for people living with motor neurone disease: A secondary analysis of people living with motor neurone disease and family member perspectives by Eleanor Wilson, Nicola Turner, Geraldine Macdonald and Christina Faull in Palliative Care and Social Practice

sj-pdf-1-pcr-10.1177_26323524261452739 – Supplemental material for Paid homecare worker support for people living with motor neurone disease: A secondary analysis of people living with motor neurone disease and family member perspectives Supplemental material, sj-pdf-1-pcr-10.1177_26323524261452739 for Paid homecare worker support for people living with motor neurone disease: A secondary analysis of people living with motor neurone disease and family member perspectives by Eleanor Wilson, Nicola Turner, Geraldine Macdonald and Christina Faull in Palliative Care and Social Practice

sj-pdf-2-pcr-10.1177_26323524261452739 – Supplemental material for Paid homecare worker support for people living with motor neurone disease: A secondary analysis of people living with motor neurone disease and family member perspectives Supplemental material, sj-pdf-2-pcr-10.1177_26323524261452739 for Paid homecare worker support for people living with motor neurone disease: A secondary analysis of people living with motor neurone disease and family member perspectives by Eleanor Wilson, Nicola Turner, Geraldine Macdonald and Christina Faull in Palliative Care and Social Practice
