# Supplementary material for: Paid homecare worker support for people living with motor neurone disease: A secondary analysis of people living with motor neurone disease and family member perspectives
Source: Palliat Care Soc Pract. 2026 Jul 4;20:26323524261452739. doi: 10.1177/26323524261452739 (PMC13333050; doi:10.1177/26323524261452739)
Supplement: sj-pdf-1-pcr-10.1177_26323524261452739 – Supplemental material for Paid homecare worker support for people living with motor neurone disease: A secondary analysis of people living with motor neurone disease and family member perspectives [file sj-pdf-1-pcr-10.1177_26323524261452739.pdf]

Understanding living with tracheostomy ventilation for Motor Neurone Disease (MND) and the implications for quality of life

Person with MND interview topic guide

Introduction

Allow time to discuss the consent form and copyright form and answer any further questions the participant might have.

- Interview involves an informal discussion; can stop at any time; no pressure to answer questions/discuss specific topics
- Can pause and restart at any time
- Permission to record
- Completion of consent to interview if not already submitted online

In this study I am interested in the experiences of people with MND who are using tracheostomy ventilation to support breathing, whether it was placed out of choice or following an emergency.

Just to get started can you tell me a bit about yourself?

- Age, employment, family
- Can you tell me about your illness? Date of diagnosis?
- How were you diagnosed? circumstances, symptoms, duration
- How have things been since then?
- Who is involved in supporting you? Family/HCPs?

How did your tracheostomy ventilation come about?

- Date of trachy operation. Did you have NIV previously?
- How did you make the decision?
- Who did you discuss it with? Who else was involved? Family/HCPs?
- What information/advice were you given to help support your decision?
- Looking back, what are your thoughts about how TV came about?
- What could have been done differently to support you with this process?

If placed in an emergency

- had you expressed any wishes/discussed this option before this happened?
- What were your initial thoughts about the TV being put in place?
- What are your thoughts about it now?

What is it like to live with TV?

- How has it benefited you? What are the drawbacks?
- How has family life been affected by the TV?
- How are you supported in your daily living?
- What care package do you have in place?
- Which HCPs are involved in supporting you with your TV? What is their role?
- Have you used any type of forum/internet group or support group to discuss living with TV?
- What might you say to other people considering TV?

What is most important to you at the moment?

- Do you have any thoughts about what might be important in the future?
- Who have you discussed this with? Family/HCPs/more widely
- Do you feel you have enough information about what might happen in the future?
- Who would you ask for more information? /discuss this with?
- Do you have any other wishes for your care in the future?
- How have you expressed these? Discussed with family / HCPs? Documented?

Is there anything about your experience of living with TV we have missed, or that you would like to add?

Debrief

Establish if the participant is experiencing any distress following the interview – extend debrief for as long as necessary to re-establish composure

End of interview. Thank participant

Understanding living with tracheostomy ventilation for Motor Neurone Disease (MND) and the implications for quality of life

Current Family Member interview topic guide

Introduction

Allow time to discuss the consent form and copyright form and answer any further questions the participant might have.

- Interview involves an informal discussion; can stop at any time; no pressure to answer questions/discuss specific topics
- Can pause and restart at any time
- Permission to record
- Completion of consent to interview if not already submitted online

In this study I am interested in the experiences of family members who are involved in the care of a person with MND who is using tracheostomy ventilation to support their breathing.

Just to get started, can you tell me a bit about yourself?

- Age, employment, family

and your family member with MND?

- Name, relationship, location
- Can you tell me about their illness? When were they diagnosed?
- How were they diagnosed, circumstances, symptoms, duration?

How did the tracheostomy ventilation come about?

- Date of trachy operation. Did they have NIV previously?
- How was the decision made? Who was involved?
- Who was it discussed with? Person with MND/Family/HCPs?
- What information/advice was given to help support the decision?
- Looking back, what are your thoughts about how TV came about?
- What could have been done differently to support this process?

What is it like to live with TV?

- What are the benefits? What are the drawbacks?
- How has family life been affected by the TV? How is [person with MND] supported with daily living?

- What care package is in place?
- What is it like for you being involved in caring for someone using TV?
- Which HCPs are involved in supporting use of TV? What is their role?
- Where do you get support - Have you used any type of forum/internet group or support group to discuss living with TV?
- What might you say to other people considering TV?

How are things for you now? What is most important to you at the moment?

- Do you have any thoughts about what might be important in the future?
- Who have you discussed this with? Person with MND/family/HCPs/more widely
- Do you feel you have enough information about what might happen in the future?
- Who would you ask for more information? /discuss this with?
- Does [person with MND] have any wishes for their care in the future?
- How have they expressed these? Discussed with family / HCPs? Documented?

Is there anything about your experience of living with TV we have missed, or that you would like to add?

Debrief

Establish if the participant is experiencing any distress following the interview – extend debrief for as long as necessary to re-establish composure

End of interview. Thank participant

Understanding living with tracheostomy ventilation for Motor Neurone Disease (MND) and the implications for quality of life

Bereaved Family Member interview topic guide

Introduction

Allow time to discuss the consent form and copyright form and answer any further questions the participant might have.

- Interview involves an informal discussion; can stop at any time; no pressure to answer questions/discuss specific topics
- Can pause and restart at any time
- Permission to record
- Completion of consent to interview if not already submitted online

In this study I am interested in the experiences of family members who were involved in the care of a person with MND who used tracheostomy ventilation to support their breathing.

Just to get started, can you tell me a bit about yourself?

- Age, employment, family, interests

and your family member who had MND?

- name, relationship
- Can you tell me about their illness?
- How were they diagnosed, circumstances, symptoms, duration?

How did the tracheostomy ventilation come about?

- How was the decision made? Who was involved?
- Who was it discussed with? Person with MND/Family/HCPs?
- What information/advice was given to help support the decision?
- Looking back, what are your thoughts about how TV came about?
- What could have been done differently to support this process?

What was it like to live with TV?

- What were the benefits? What were the drawbacks?
- How was family life affected by the TV?
- How was [person with MND] supported with daily living?

- What care package was in place?

- What was it like for you being involved in caring for someone using TV?
- Which HCPs were involved in supporting use of TV? What was their role?
- Have you used any type of forum/internet group or support group to discuss living with TV?
- What might you say to other people considering TV?

What were the circumstances of [person with MND's] death?

- Location, timing?
- What events led up to their death?
- Did you feel you had enough information about what might happen?
- Who did you discuss this with?
- Did [person with MND] have any wishes for their care towards the end of life?
- How did they express these? Discussed with family / HCPs? Documented?

How are things for you now? What is most important to you at the moment?

Is there anything about your experience of living with TV we have missed, or that you would like to add?

Debrief

Establish if the participant is experiencing any distress following the interview – extend debrief for as long as necessary to re-establish composure

End of interview. Thank participant
