# Supplementary material for: Paid homecare worker support for people living with motor neurone disease: A secondary analysis of people living with motor neurone disease and family member perspectives
Source: Palliat Care Soc Pract. 2026 Jul 4;20:26323524261452739. doi: 10.1177/26323524261452739 (PMC13333050; doi:10.1177/26323524261452739)
Supplement: sj-pdf-2-pcr-10.1177_26323524261452739 – Supplemental material for Paid homecare worker support for people living with motor neurone disease: A secondary analysis of people living with motor neurone disease and family member perspectives [file sj-pdf-2-pcr-10.1177_26323524261452739.pdf]

Exploring end of life decision making with patients with Motor Neurone Disease (MND) using home mechanical ventilation: The perspectives of PwMND.

Person with MND interview topic guide

Introduction

- *Introduction to researcher and study*
- *Interview involves a free and informal discussion; confidential; can stop at any time; no pressure to answer questions/discuss specific topics*
- *Can pause and restart at any time*
- *Permission to record*
- *Completion of consent to interview*

In this study I am interest in the experiences of people with MND who are using ventilation at home to alleviate the symptoms of MND and can no longer effectively breathe without it (dependent)

Just to get started can you tell me a bit about yourself?

- Age, employment, family, interests
- Can you tell me about your illness?
- How were you diagnosed? circumstances, symptoms, duration
- How have things been since then?
- Who is involved in supporting you? Family/HCPs?

When did you decide to use ventilation?

- How did you make the decision?
- Who did you discuss it with? Who else involved? Family/HCPs?
- What information were you given?
- Was there any discussion about your use of ventilation in the future?
- Has it been discussed since?
- What do you think about the timings of the discussions you have had?
- Have you used any type of forum/internet group or support group to discuss these issues?

What are your thoughts about the use of ventilation in the future?

- Who have you discussed these with? Family/HCPs/more widely
- Do you have any other wishes for your care in the future?
- How /have you expressed/documented these? Discussed with family?

Do you feel you know enough about what is likely to happen as you become increasingly ill?

- What else would you like to know about? Who to ask?
- Role of family

Anything else, missed, not discussed?

Establish if experiencing any distress as a result of the interview – extend debrief for as long as necessary to re-establish composure

End of interview and Thanks!

Exploring end of life decision making with patients with Motor Neurone Disease (MND) using home mechanical ventilation: The perspectives of families.

Bereaved Family Member interview topic guide

Introduction

- *Introduction to researcher and study*
- *Interview involves a free and informal discussion; confidential; can stop at any time; no pressure to answer questions/discuss specific topics*
- *Permission to record*
- *Completion of consent to interview*

In this study I am interest in the experiences of family members who have experienced bereavement after being involved in the care of someone dependent (using ventilation for 16hrs or more per day) on home ventilation to alleviate the symptoms of MND.

First of all: can you tell me a bit about yourself and your family?

- [names] illness – symptoms, diagnosis, circumstances, duration, place of care
- relationship with them? involvement in support, care and extent of contact?
- Other key people involved – their role

Ventilation:

- What was it like being involved in the care of someone using home ventilation?
    - Who else was involved in that care?
- How was the decision made to start?
    - Who/how discussed, information given
    - When was the role of ventilation in the future discussed? - thought about that in advance?
- Awareness and communication about dying and anticipated death within the family and between the family and 1. dying person 2. HCPs
- wishes regarding their treatment or care
    - thoughts about preferences to stop or continue ventilation
    - Who did they/[name] talk to?
    - Agreement/disagreement
    - Did [name] have any other wishes? - was it possible to fulfil these wishes?

The last few days:

- reflection on ‘quality’ of experience: medical management, control of symptoms, distress, decision making, family involvement in decision making
    - positive aspects of the experience
    - negative aspects/regrets – things that might have been otherwise
    - as expected
    - what support given/needed
    - wider impacts – family/friends/work/social

Key messages:

- for other families/patients/ HCPs – info needed

Anything else, missed, not discussed?

Establish if experiencing any distress as a result of the interview – extend debrief for as long as necessary to re-establish composure

End of interview and Thanks!

Exploring end of life decision making with patients with Motor Neurone Disease (MND) using home mechanical ventilation: The perspectives of families.

Current Family Member interview topic guide

Introduction

- *Introduction to researcher and study*
- *Interview involves a free and informal discussion; confidential; can stop at any time; no pressure to answer questions/discuss specific topics*
- *Permission to record*
- *Completion of consent to interview*

In this study I am interest in the experiences of family members who are involved in the care of someone dependent (using ventilation during the day as well as at night) on home ventilation to alleviate the respiratory symptoms caused by MND.

First of all: can you tell me a bit about you and your family?

- [name] illness – symptoms, diagnosis, circumstances, duration, location
- relationship with them? involvement in support, care and extent of contact?
- what is it like being involved in caring for someone using HV
- other key people involved – their role

Ventilation:

- how was the decision made to start?
  - Who/how discussed, information given
  - Was future use also discussed? - thought about that in advance?
- wishes regarding their treatment or care
  - thoughts about preferences to stop or continue ventilation
  - have these been discussed
  - Agreement/disagreement
  - Does [name] have any other wishes? /wishes for them
- Awareness and communication about dying and anticipated death within the family and between the family and 1. dying person 2. HCPs
- Do you feel you have enough information about what will happen in the future?
  - Who would you ask for more information?/discuss this with?

The decision:

- reflection on ‘quality’ of decision making: place in decision making process, information, support, family involvement, communication
    - review of the decision – how/by whom/ recorded
    - positive aspects /negative aspects/what could be done differently
    - what support given/needed
    - concerns for future/fear/worries
    - wider impacts – family/friends/work/social

Key messages:

- for other families/patients/ HCPs – info needed

Anything else, missed, not discussed?

Establish if experiencing any distress as a result of the interview – extend debrief for as long as necessary to re-establish composure

End of interview and Thanks!
